# The recurrence of the melanotic neuroectodermal tumour of infancy: an unusual presentation of a rare tumour

**DOI:** 10.3332/ecancer.2020.1049

**Published:** 2020-05-26

**Authors:** Antonio Augusto Claudio Pereira, Mayara Maria de Jesus Rozante, Rafael Bauer Doveinis, Carmine Porcelli Salvarani, Tania Hissa Anegawa, Paola da Costa Souza, Daniel J Brat, Alessandra Cristina de Oliveira Borges

**Affiliations:** 1 University Center of Maringá, Maringá, PR 87050-390, Brazil; 2 Marilia Medical School, Marília, SP 17519-030, Brazil; 3 Neurosurgery department, Holy House of Mercy of Maringá, Maringá, PR 87050-100, Brazil; 4 State University of Londrina, Londrina, PR 86039-440, Brazil; 5 State University of Maringá, Maringá, PR 87083-240, Brazil; 6 Northwestern University Feinberg School of Medicine, Chicago, IL 60611, USA; a https://orcid.org/0000-0001-9808-8052; b https://orcid.org/0000-0002-6797-7700; c https://orcid.org/0000-0002-1483-9128

**Keywords:** case report, melanotic neuroectodermal tumour of infancy, melanotic progonoma, skull

## Abstract

The melanotic neuroectodermal tumour of infancy (MNTI), also known as melanotic progonoma, is a rare neoplasm derived from neural crest cells. Although it is fundamentally benign, the tumour may present a locally aggressive behaviour, characterised by a rapid progression and a destructive invasion of adjacent structures, hence causing deformities. Unfortunately, perhaps due to the low incidence of this type of tumour, the published cases in the literature do not characterise the factors that imply the malignant or recurrent behaviour of the disease, nor the therapy to conduct these cases. Here, we report a rare case of a recurrent benign MNTI, approached unusually with a favourable outcome.

## Introduction

The melanotic neuroectodermal tumour of infancy (MNTI) is a rare neoplasm derived from neural crest cells [1]. Before ultrastructural, immunohistochemical and immunoelectron microscopic studies, the odontogenic or retinal remains were the suspected origin of the MNTI [2]. It was called in the old nomenclature by many different terms, such as retinal anlage tumour, pigmented congenital epulis, melanotic progonoma, melanotic adamantinoma [1,3,4] or congenital melanoma—the latter was its first designation when described by Krompecher [3]. Elevated urinary excretions of vanillylmandelic acid (VMA), identifiedby Borello and Gorlin [5], were fundamental for the understanding of the histopathology of the tumour since there was a suspected origin of the neural crest [2].

The tumour usually occurs in infants, with a male predilection and mainly between the age of 2 and 6 months, with the head and neck as the most affected regions [1, 2, 4, 6, 7]. The investigation is based on the identification of a painless craniofacial mass, with progressive growth and without neurological impairment, which in some cases can cause seizures, increased intracranial pressure, acute hydrocephalus and neurological dysfunction [8]. It may be present since birth or it may appear sporadically. Occasionally, it can be an accidental radiological finding [4]. Histologically, it is characterised by the formation of areas with small globular cells and areas with large polygonal cells containing melanin, which combine neural cells, melanocytes, and epithelial cells [1, 3].

Although it is a fundamentally benign neoplasia, the tumour may present a locally aggressive behaviour [4, 9] characterised by a rapid progression and a destructive invasion of adjacent structures, hence causing deformities [8]. The recurrence rate is roughly from 20% to 25% [8, 10], with malignant transformation, and metastasis in 6.5% of all the cases [10]. Unfortunately, perhaps due to the low incidence of this type of tumour, the published cases in literature do not characterise the factors that imply the malignant or recurrent behaviour of the disease, nor the therapy to conduct these cases [6]. Here, we report a rare case of a recurrent benign MNTI, approached unusually with a favourable outcome.

## Case description

A 1-year-old boy was referred for evaluation by the neurosurgery team with an expansile nodular lesion in the right retroauricular region. The mother stated that a retroauricular bulging has been present in this region since his birth; however, the professionals who were consulted at the time believed there was no clinical significance. The neuropsychomotor development (NPMD) of the child was normal for his age and he had no previous significant history of pathology. His maternal uncle was diagnosed with acute myeloid leukaemia at the age of 9. Germline genetic testings were not performed.

**Figure 1.**
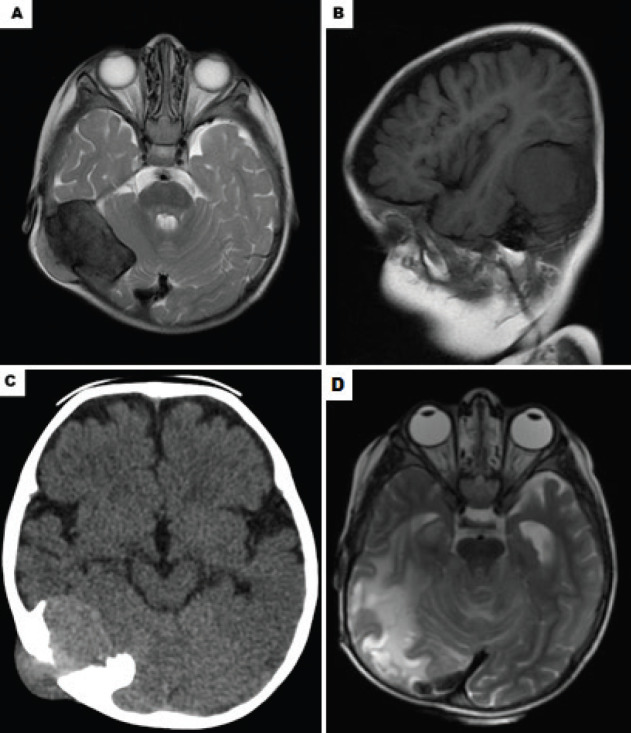
Imaging exams highlighting the injury of the patient. (A): Axial contrast-enhanced CT scan showing neoplastic lesion with an epicentre on the skullcap of the right temporo-occipital transition with intracranial and extracranial expansion. (B): Sagittal contrast-enhanced CT scan showing the same lesion. (C): Magnetic resonance imaging shows a highly enhancing tumour with epicentre in the right side of the posterior cranial fossa. (D): Computerizsd tomography 3 years after the last surgery, the patient accompanied only with chemotherapy and radiotherapy.

The initial skull radiography showed bone sclerosis associated with probable intracranial calcification in the right retroauricular region at the parieto-occipital transition. The ultrasound highlighted a homogeneous and regular hypoechoic nodule, with 32 × 26 × 12 mm, located below the subcutaneous tissue in close contact with the adjacent bone cortex. The colour Doppler imaging identified peripheral vessels adjacent to the main lesion. Computerised tomography revealed a 4.2 × 4.5 × 3.6 cm of a neoplastic lesion with an epicentre on the skullcap of the right temporo-occipital transition with intracranial and extracranial expansion; the lesion was compressing the adjacent cephalic structures apparently in a supratentorial compartment (Figure 1A and B). Brain magnetic resonance displayed an intracranial component in the posterior cranial fossa, compressing the adjacent cerebellar hemisphere. In the middle cranial fossa, the component was rectifying the turns of the posterior aspect of the anterior temporal pole on this side (Figure 1C). Among the diagnostic hypotheses were the fibrosarcoma, the meningioma, and the eosinophilic granuloma.

**Figure 2.**
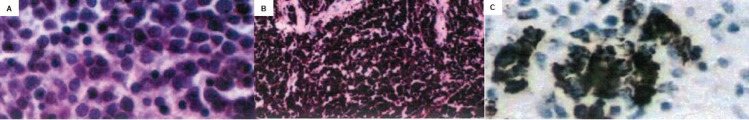
Anatomopathological and immunohistochemical findings. (A): Hematoxylin and eosin stains show neoplasm with a biphasic pattern characterised by pseudoglandular hyperchromatic cells associated with dense fibrovascular stroma. (B): Synaptophysin was positive in immunohistochemistry. (C): Focal Positivity for gp100 Antigen in Immunohistochemistry.

The patient underwent surgery and intraoperative frozen section. The histopathology of the tissue revealed a biphasic tumour tangential to the resection margins, characterised by small, round and blue cells, as well as pseudoglandular arrangement with pigmented cells infiltrating the bone and muscle tissue (Figure 2A). We initially concluded that it was a MNTI, but due to the rarity of the diagnosis, the material was sent for an immunohistochemical study in a reference laboratory.

The immunohistochemical study presented expression of Ki-67, synaptophysin (Figure 2B), product of the INI-1 gene and 40, 48, 50 and 50.6 kDa cytokeratin, being the antigen gp100 focally positive (Figure 2C). Besides that, glial fibrillary acidic protein (GFAP), desmin, myogenin, S-100 protein and homeobox protein (transcription factor gene, clone 74.5A5) were negative. The diagnosis of medulloblastoma with melanocytic differentiation (grade IV, WHO) was suggested. However, after a review at the Neuropathology Service of Emory University, in Atlanta, USA, the diagnosis of MNTI was established.

Three months after the procedure, the lesion relapsed at the primary site and a second surgery was performed with complete removal of the tumour. After 2 months, seizures started to evolve in the patient and a new relapse was observed. A brain and cervical spine MRI demonstrated a heterogeneous and expansive neoplastic lesion with an epicentre on the right side of the posterior cranial fossa, which showed an increase in the dimensions (5.4 × 5.2 × 4.3 cm) when compared to previous exams. This determined a compression on the cerebral aqueduct causing obstructive hydrocephalus and signs of obstructive hydrocephalus with transependymal cerebrospinal fluid flow, requiring a new neurosurgical procedure.

The postoperative revaluation revealed the persistence of the extracranial component in the cervical region with a slight increase in its dimensions, appearance of the cavitation area and necrosis. MRI revealed an increase in the number and extension of the right cervical lymph nodes. Posteriorly, the patient underwent a new surgery for tumour excision.

Afterward, the child was referred for assessment by the paediatric oncology team. Although it is considered to be a benign pathology, chemotherapy treatment was proposed due to the relapse and limitations of the new surgical approach. Six cycles of the chemotherapy were performed with a 21-day interval. First cycle: Vincristine 1.5 mg/m^2^/dose, Doxorubicin 30 mg/m^2^, Cyclophosphamide 600 mg/m^2^/day (VAC); Second to sixth cycle: Cyclophosphamide 900 mg/m^2^/dose, Vincristine 1.5 mg/m^2^/dose, Carboplatin 450 mg/m^2^, evolving with an apparent resolution of lesions.

Unfortunately, the patient presented a local recurrence (in the craniectomy region) 5 months after the end of the treatment, so a new surgery was performed. The morphological and immunohistochemical patterns were compatible with the recurrence of MNTI and, therefore, were chosen to restart the chemotherapy based on the high-risk sarcomas protocol of the hospital.

Despite the treatment, the patient developed leptomeningeal dissemination, which was manifested by the motor deficit, walking difficulties, inability to stand and seizures that were difficult to control. We chose to use palliative 3D conformal radiotherapy (skull + spine) (4,350 cGy + boost 1,800 cGy). The patient presented an improvement of the neurological symptoms with the recovery of the motor skills and the control of seizures. The expectant management with radiological control of the lesions was adopted.

After 3 years, control imaging exams revealed signs of supratentorial and infratentorial leptomeningeal impregnation, suggesting a postoperative scar (Figure 1D). Outpatient follow-up was selected since the patient remained asymptomatic and there is no literature supporting other therapeutics.

The presentation of this case was authorised by the mother of the child, as well as by the Ethics and Research Committee of the Hospital approached in this study.

## Discussion

The MNTI is a rare benign tumour primarily located in the maxilla (60.3%), followed by the skull (18.1%) and the mandible (10.3%), the remaining incidence is on the epididymis, testis and brain [10]. Within the possible sites of cranial involvement, the parieto-occipital transition in the retroauricular region is the most frequently involved site, present in 54.9% of the cases, as in the patient of this study [8].

Studies state that the diagnosis of MNTI is made before the 12 months of age [12], specifically with the cranial incidence, having a diagnosis with an average age of 16.4 months [8]. Thus, there was no obvious delay in the diagnosis of the case-patient. Suggestive radiological features of this tumour include a diameter superior to 5.4 cm [8], presence of a regular, hypoechoic, circumscribed pattern and bone sclerosis areas [9], which resemble the patterns described herein. Although imaging studies contribute to the tumour investigation, they are unable to confirm the diagnosis of the disease. The fact that there are only a few studies in the literature relating the tumour with radiological findings may complicate or confuse the investigative process [3].Regarding biological markers, there was no study of metabolites in this patient, once their usage is questionable in the literature. The VMA can be defined as a tumour marker since its urinary levels decrease after tumour resection [13]; however, it is not essential to define conducts, because even though the disease is present, the urinary analysis can be negative [14, 15]. No molecular tests were performed on the tumour tissue sample, except for immunohistochemical markers. Some studies have attempted to find genetic changes present in the MNTI—1p exclusion, a gain in 7q and MYC [16–18]—common to other tumours with a greater description, such as neuroblastomas. In addition, a presence of similar histological characteristics—neuroblastoma cell types—justifies the influence on the direction of the treatment. However, these studies are scarce and present conflicting results, some of them show neuroblastoma-like alterations [16], and others did not show the presence of neuroblastoma-related mutations [17].Hence, for an accurate and definitive diagnosis, the histological study should be performed. In this patient, the histological characteristics were consistent with those described in the literature, with the presence of small, round and pigmented cells, thus helping in the exclusion of other differential pathologies [12]. The same is true for immunohistochemistry when the MNTI is associated with synaptophysin and cytokeratin [2]. Nonetheless, there are still controversies that might be an obstacle to an accurate diagnosis [15]. In this case, the initial analysis suggested a medulloblastoma with melanotic differentiation, requiring subsequent investigation to confirm MNTI.

Although most studies present the MNTI as a benign tumour, about 25% of the cases in the cranial region have a recurrence [7]. A recently published systematic review [8] analysed 91 cases of brain and skull MNTI from the last 100 years, encountering only 20 individuals with a recurrence of the disease, such as the case shown in this report. The recurrent behaviour was significantly associated with age, tumour size (larger diameter superior to 5 cm), resection without margin excision, metastasis and malignancy, which is mainly observed in patients with cerebral MNTI and older age for diagnosis. In this study, we describe six recurrences that occurred within less than 6 months between the procedures, similar as presented by the author Santos [9]; however, the case described here does not share these aforementioned risk characteristics, which makes the recurrent behaviour more unexpected.

The first line of treatment for MNTI is known to be a complete surgical resection with a tumour-free margin, and if it is a benign neoplasm, it is usually curative. In cases of malignancy, metastasis can occur in the regional lymph nodes, liver, and bone marrow and is associated with a high mortality rate. The chemotherapy is an alternative to slow the progression of the disease [19]. The key point of the discussion occurs in tumours with benign historical characteristics, but with a recurrent behaviour and local aggressiveness, as in this case. In this scenario, the association of chemotherapy and radiotherapy as adjunctive therapies remains controversial and literature is scarce regarding this subject. Our case demonstrates that in situations in which the surgical approach becomes limited, this association may be beneficial for the patient prognosis. Among the 20 individuals with recurrent disease identified by Ren [8], only one patient, described by Furtado [20], underwent a combination of surgery, chemotherapy and radiotherapy for adequate tumour control, as addressed in this study. However, the case described [20] was a malignant MNTI, a fact that guided the use of the combined therapy.

We have not found data to support the management of widely recurrent cases, such as the one described herein. Although this tumour has benign histological characteristics, the surgical approach was limited and with progressively greater risks due to the several recurrences.

Other authors describe associations with chemotherapy and radiotherapy regimens, but without an established protocol, differing between the moment and the scheme employed for each patient [16, 19, 21]. The challenge in managing cases such as the one in this report, in which the most commonly used therapy—surgery—becomes unfeasible, is due to the lack of a pattern in the administered regimens, most of them established on empirical administration based on therapies for other types of tumour. The most commonly used regimens are combinations of drugs studied for the treatment of neuroblastomas—vincristine, ifosfamide, carboplatin and/or Endoxan—due to the presence of some histological similarities [20, 22]. Some studies show a more aggressive approach, with early use of adjuvant therapies [15]. In this case, a milder and shorter method was initially chosen, requiring a more aggressive approach with the use of chemotherapy after the recurrence. Only after the unsuccessful attempts to control the disease, radiotherapy was utilised as a therapeutic alternative, obtaining favourable results. From this development, it is suggested that radiotherapy may be superior to chemotherapy since the tumour presented resistance to the administered drugs.

## Conclusion

This case highlights the difficult process of diagnosis and management of an uncommon presentation of MNTI, presenting a favourable outcome despite the recurrent behaviour. We find that the absence of data, which contributes to a further discussion about the therapy, is a limitation to more favourable development of the case, once the disease debated herein does not have an established protocol.

Although the therapeutic scheme utilised in this case was empirical and distinct from the common treatment for benign tumours, the fact that we observed a favourable outcome may have contributed to the guidance of the management of recurrent and challenging cases. We suggest the early administration of the adjuvant therapies in patients presenting MNTI with more aggressive behaviour (two or more recurrences) and/or characteristics of greater risk (brain injury, diameter superior to 5 cm, resection without excision margin). In patients over 5-year old, radiotherapy would be our first choice, as it has been showing to be more effective than chemotherapy. However, due to the risk of sequelae in younger patients in the NPMD, the alternative would be chemotherapy. We emphasise that therapies should be administered as adjuvants, with the surgical procedure remaining as the main pillar of the treatment.

New research that contributes to a better understanding of this pathology and helps with the management of affected patients is necessary for superior therapeutic options for patients.

## Informed consent

The mother of the patient gave her informed consent for the publication of this case.

## Conflicts of interest

The authors have no conflicts of interest.

## Funding

There is no financial support for this report.
